# Comparative host–graft biomechanics of osteoarticular allografts and allograft–prosthesis composites after distal femoral tumor resection

**DOI:** 10.1186/s13018-026-07127-1

**Published:** 2026-08-01

**Authors:** Vasileios Apostolopoulos, Petr Boháč, Michal Mahdal, Luboš Nachtnebl, Tomáš Návrat, Tomáš Tomáš

**Affiliations:** 1 First Department of Orthopaedic Surgery, St. Anne’s University Hospital and Faculty of Medicine, Masaryk University, Brno, Czechia; 2 Institute of Solid Mechanics, Mechatronics and Biomechanics, Faculty of Mechanical Engineering, Brno University of Technology, Brno, Czechia

## Abstract

**Background:**

Distal femoral reconstruction after tumor resection can be performed using osteoarticular allografts (OCH) or allograft–prosthesis composites (APC). Although both techniques restore bone stock, complications such as nonunion, graft fracture, and hardware failure remain frequent. These events are closely related to the mechanical environment at the host–graft junction, yet direct biomechanical comparison under identical conditions is limited. Therefore, this study aimed to compare the mechanical behavior of OCH and APC reconstructions following distal femoral tumor resection using finite element analysis, focusing on graft strain, hardware stress, and interfragmentary motion (IFM).

**Methods:**

Two patient-specific finite element models were created from CT data, simulating a 150-mm distal femoral defect reconstructed either with a plate-fixed osteoarticular allograft (OCH) or with a cemented revision rotating-hinge allograft–prosthesis composite (APC). A 400 N axial load was applied to represent early postoperative weight bearing. Equivalent strain, von Mises stress, IFM, and sliding distance at the host–graft interface were quantified.

**Results:**

OCH demonstrated higher peak graft strain (5700 µε vs. 3200 µε) and increased IFM (median 6.5 μm vs. 2.5 μm) compared with APC. Hardware stress was 77 MPa in OCH and 24 MPa in APC. Sliding distance was also greater in OCH (3.5 μm vs. 0.6 μm). APC showed reduced graft strain and micromotion consistent with load redistribution through the prosthetic stem and cement mantle.

**Conclusion:**

Under identical geometry and early postoperative loading conditions, OCH and APC reconstructions demonstrated distinct load-transfer patterns. OCH was associated with greater graft strain and micromotion, whereas APC demonstrated lower graft strain and greater construct rigidity. Rather than indicating superiority, the findings highlight that each reconstruction is governed by a characteristic mechanical environment that may influence its failure pattern.

## Background

The distal femur is the most frequent location of primary bone tumors, particularly in children [1, 2]. Although wide resections are often required, the current trend is toward limb-salvage procedures with reconstruction of the resulting bone defect. Reconstructing large defects around the knee after tumor resection remains a significant challenge. Contemporary reconstruction options include osteoarticular allografts, modular megaprostheses, and allograft–prosthesis composites (APC) [3–5]. When osteoarticular allografts or allograft–prosthesis composites are used, bone stock is restored, preserving the potential for multiple revision strategies in the long term [6–8].

Osteoarticular allografts (OCH) have been associated with high early complication rates, particularly mechanical failures such as nonunion and graft fracture, as well as early onset of knee osteoarthritis [7, 9]. The APC, in which a revision prosthesis is implanted into a structural bone allograft, was developed to address these shortcomings by combining the biological advantages of the allograft with the mechanical reliability of the prosthesis [10]. While earlier reports on APCs described relatively high complication rates, more recent use of frozen allografts in combination with rotating-hinge prostheses has yielded more promising clinical outcomes [11, 12]. Nevertheless, both techniques share persistent problems at the host–graft junction, where nonunion, resorption, and structural failure remain common [10]. These complications are closely linked to the local mechanical environment, particularly micromotion and stress distribution at the interface, yet the underlying mechanics are not fully understood and have not been systematically characterized in a comparative manner between osteoarticular allografts and APC constructs. Importantly, OCH and APC differ not only in implant configuration, but also in their basic fixation principle. OCH relies on eccentric plate fixation with load sharing through the structural allograft, whereas APC uses central stem fixation with partial replacement of cancellous bone by the prosthetic stem and cement mantle. These differences may influence the mechanical environment relevant to host–graft healing and therefore represent an inherent part of the construct-specific comparison addressed in this study.

Given that these complications are fundamentally driven by local mechanical conditions at the host–graft junction, a numerical approach capable of resolving three-dimensional stress, strain, and micromotion patterns is required. Clinical series can document the incidence and timing of nonunion, graft resorption, and structural failure, but they cannot directly reveal the underlying mechanical environment, and in vitro experiments are limited by simplified geometries, small sample sizes, and difficulties in instrumenting internal interfaces [4, 7]. Finite element analysis overcomes many of these limitations by allowing realistic reconstruction of distal femoral anatomy and implant–allograft configurations, assignment of material properties to bone, implants, and application of physiologic loading conditions representative of daily activities [13]. In this way, finite element modeling provides a powerful framework to systematically compare osteoarticular allograft and APC constructs, quantify interface micromotion and junctional stress distributions [14, 15].

Building on these clinical and biomechanical considerations, the rationale of the present study was to isolate and compare the mechanical environment created by osteoarticular allografts and APCs in an otherwise identical distal femoral defect. By constructing two finite element models with the same bone defect and host anatomy—one reconstructed with an osteoarticular allograft and the other with an APC using a revision rotating-hinge design—we aimed to directly quantify differences in load transfer, stress distribution within the structural allograft, and micromotion at the host–graft junction. This controlled numerical comparison allows us to test whether the APC configuration, which clinically appears to reduce some graft-related complications, indeed provides a more favorable mechanical environment for junctional union and long-term graft integrity, or whether it simply shifts the location and type of mechanical risk. Through this approach, our aim was to provide mechanistic insight that clarifies the clinical failure patterns of both reconstructions and guides the surgical technique for distal femoral tumor reconstruction.

## Methods

### Geometry models

Two CT scans of the lower limb were obtained using a CT scanner (GoldSeal Optima CT660, GE HealthCare, CI, USA) with a voxel size of 0.9336 × 0.9336 × 0.625 mm3. In a custom application developed in the MATLAB 2012 (MathWorks, Natick, MA, USA) environment, the CT data were processed to reconstruct the 3D geometry of the femur and prepare it for finite element analysis [16]. The CT scans were manually segmented to generate a Standard Tessellation Language (STL) file containing the surface geometry of two femurs and tibiae. Using a 3D scanner (Shining3D EinScan SE, SHINING 3D Technology GmbH, Stuttgart, Germany), STL format files of Beznoska revision rotating-hinge knee prosthesis (Beznoska s.r.o., Kladno, Czech Republic) components were obtained.

The geometry models of the distal femoral plate (DFP) and corresponding screws were obtained directly from the manufacturer (MEDIN, a.s., Nové Město na Moravě, Czech Republic). The DFP measures 299 mm in length, with 12 holes in the proximal region and a thickness of 6 mm (cat. no. 397129779620). Subsequently, all STL files were modified and converted to solid geometry in SpaceClaim ANSYS® Academic Research Mechanical, Release 25.2 (Swanson Analysis, Inc., Houston, PA, USA). The geometry of the DFP was modified by substituting the internal threads with conical surfaces. The same modification was applied to the screw head [17, 18].

**Fig. 1:**
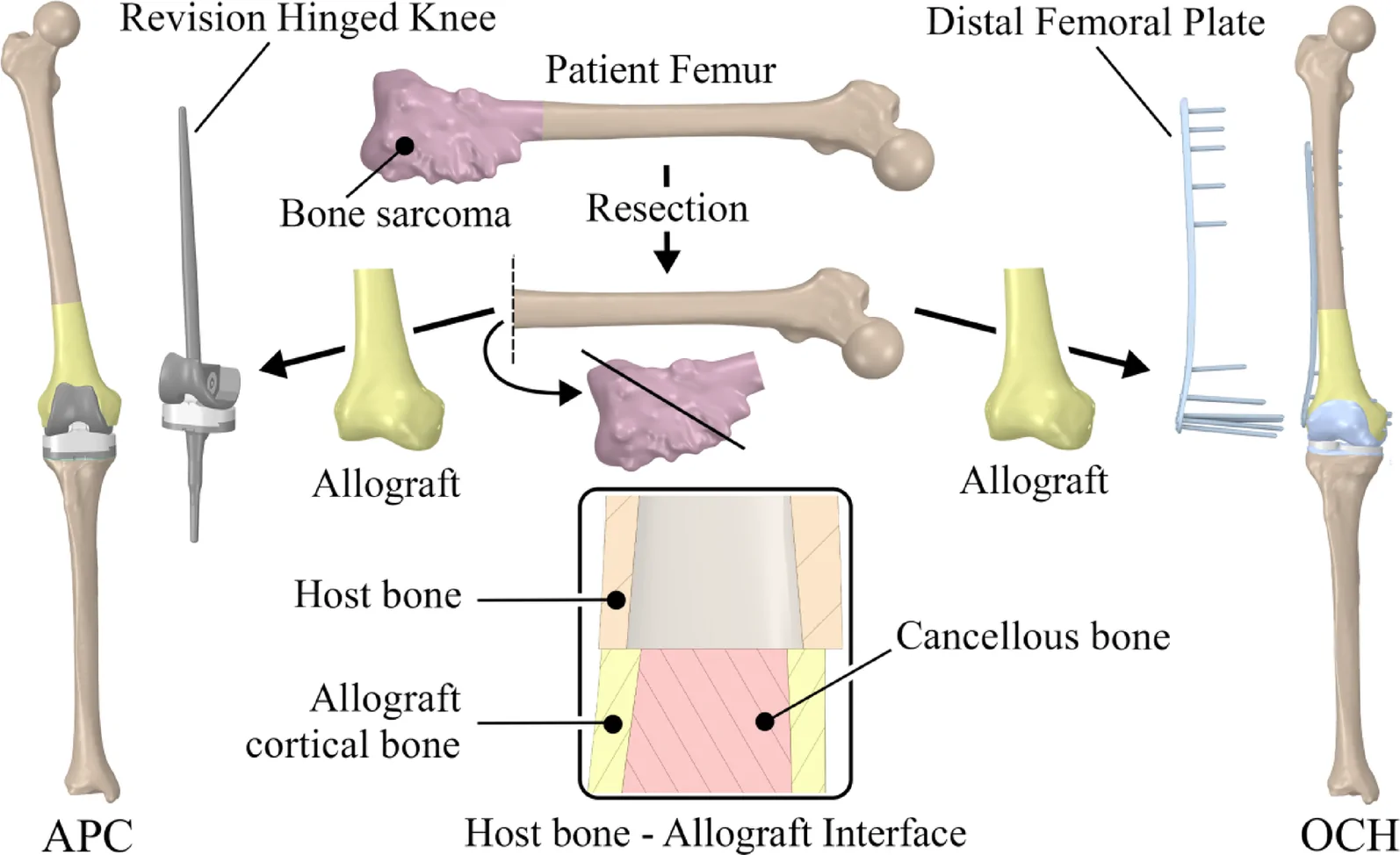
Resection of bone sarcoma in femoral model A, followed by defect reconstruction using an allograft from femoral model B, and creation of OCH and APC geometry with sectional interface detail to OCH model

The femur bone tissue models were created from CT scans of two patients, labeled “A” and “B.” Model “A” was created from CT scans of a representative patient diagnosed with a bone tumor of the femur. Model “B” was created from CT scans of a healthy individual. The bone size corresponds proportionally to Model “A”, ensuring geometric consistency between the two reconstruction scenarios while maintaining comparable anatomical dimensions. A 150 mm long part in the area of the distal femur with a tumorous lesion was removed from the femur “A” model to simulate the surgery process. The defect in the femur “A” model was replaced with a corresponding part of the same length from the femur “B” model to simulate an OCH reconstruction of the distal femur. In the second model, the defect in the femur “A” model was replaced with a corresponding part of the same length from the femur “B” model, including revision rotating-hinge knee prosthesis to simulate an allograft–prosthesis composite (APC) reconstruction of the distal femur, as shown in Fig. 1. The bone geometry and material properties distinguish cortical and cancellous bone tissues.

**Fig. 2:**
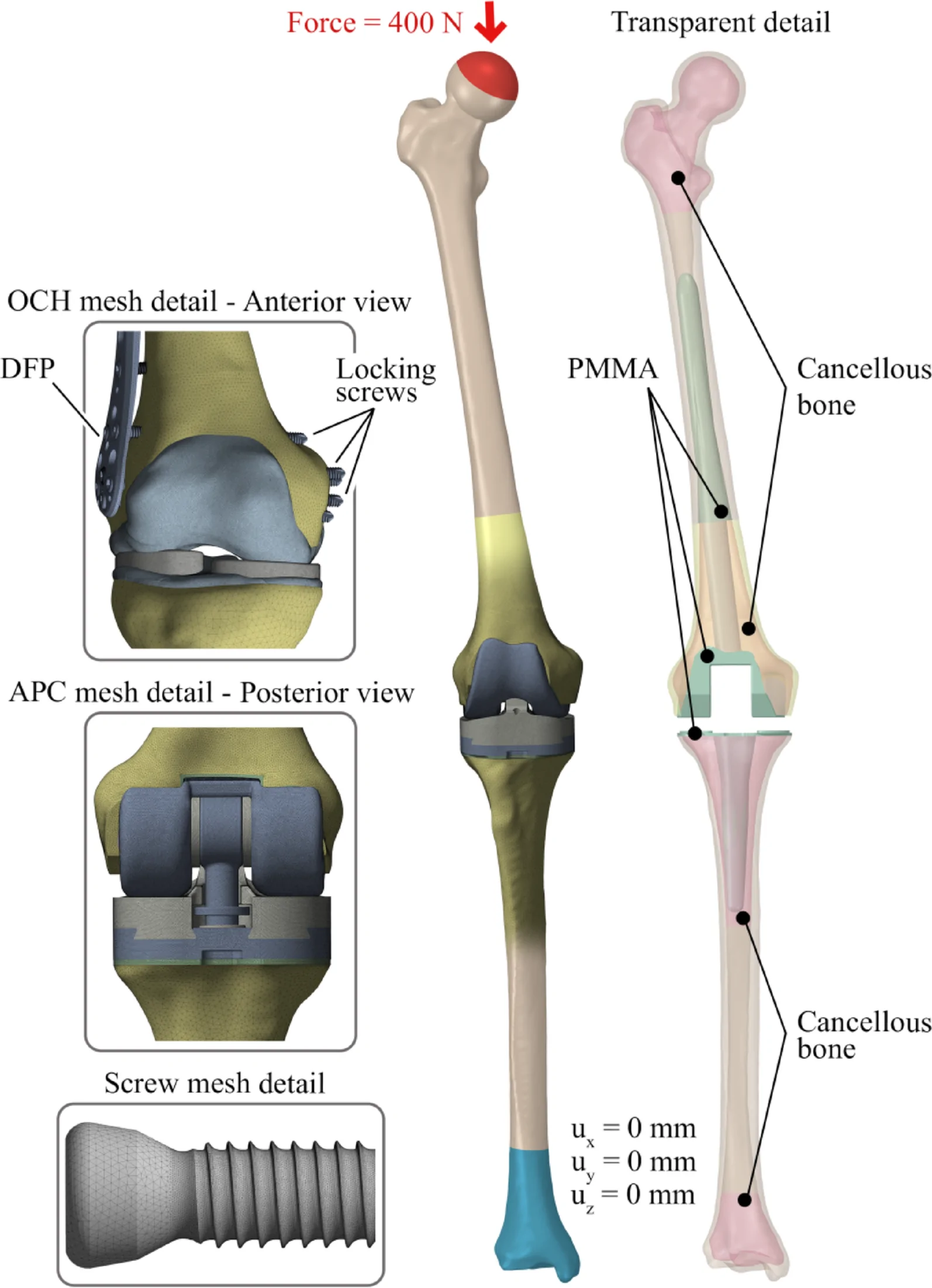
APC reconstruction geometry model with boundary conditions and component labels. Transparent view revealing cancellous bone and PMMA geometries, plus mesh details for knee reconstruction (both models) and screw

In the OCH reconstruction model, one DFP and 12 locking bone screws (HA 5), each with a diameter of 5 mm, but a variable length of 30–95 mm (standardized lengths graduated in 2 or 5 mm increments), were added to this assembly according to the principles of Arbeitsgemeinschaft für Osteosynthesefragen (AO) [19]. In addition to the OCH model, models of the tibia (patient A), articular cartilages, and menisci were included. Their geometries and dimensions were modeled based on anatomical data, with local thicknesses maintained within physiologically appropriate ranges. The modeling approach followed established methods from published studies that provide detailed descriptions of meniscal and cartilage geometry [20, 21]. In the APC model, a revision rotating-hinge knee prosthesis was implanted to replicate the standard reconstruction of a defect identical to that used in the OCH model. Polymethylmethacrylate (PMMA) bone cement was applied to simulate cemented fixation of the APC construct. In the allograft part, the thickness of the PMMA was uniformly set to 1 mm. In the original bone and tibial parts, the PMMA thickness corresponds to the residual void created by drilling with a reamer approximately 2 mm larger in diameter than the stem, representing the intended surgical cement space (1 mm radial clearance on each side), as illustrated in Fig. 2. This uniform cement mantle assumption is an idealization adopted to ensure model reproducibility and avoid introducing additional geometric bias.

### Surgical technique

In distal femoral tumor reconstruction using an APC with a revision rotating-hinge knee prosthesis, wide tumor resection is first performed to achieve oncologically safe margins while preserving adequate host (original) bone for fixation. A size-matched fresh-frozen structural distal femoral allograft is selected and prepared on the back table, including canal reaming and shaping to accommodate the revision stem. The revision rotating-hinge femoral component is then cemented into the allograft using PMMA, ensuring correct alignment, rotation, and stem depth to create a stable composite construct. The host femoral canal is sequentially prepared, and the APC is inserted, typically with a long intramedullary stem that may be cemented or press-fit into the host bone, depending on bone quality and surgical preference.

In distal femoral reconstruction using an osteoarticular allograft, wide tumor resection is performed with adequate oncologic margins. A size-matched fresh-frozen distal femoral osteoarticular allograft is selected and shaped to reproduce the resected segment. The allograft is inserted and rigidly fixed to the host femur using a long stainless-steel locking plate (DFP) with multiple bicortical screws placed proximally and distally to achieve stable fixation at the junction. After osteosynthesis, ligamentous reconstruction is performed to restore knee stability. The anterior cruciate ligament (ACL), medial collateral ligament (MCL), and lateral collateral ligament (LCL) are reconstructed using Trevira® (polyethylene terephthalate) soft tissue reconstruction material [22]. The Trevira grafts are passed through anatomically positioned bone tunnels in the allograft and proximal tibia, or secured to the corresponding epicondylar regions, and fixed with interference screws or cortical fixation devices under appropriate tension.

### Meshing procedure

All solid bodies were discretized in ANSYS® using second-order hexahedral (SOLID186) and second-order tetrahedral (SOLID187) elements. Contact interfaces were modeled with CONTA174 contact elements paired with TARGE170 target elements. The resulting meshes contained approximately 20 million nodes for the OCH model and 14 million nodes for the APC model. Global element sizes were set to 1.5 mm for cortical bone and 3 mm for cancellous bone. All surfaces of the revision rotating-hinge knee prosthesis were meshed with an element size of 0.5 mm; the PMMA, cartilage, and meniscus surface layers were also assigned 0.5 mm. The DFP was discretized with a 1 mm element size. Screws were meshed with local refinement ranging from 0.05 ${\mathrm{mm}}$ at the threads to 1 mm in the shank and global regions. The above-mentioned element sizes were determined through preliminary tests and sensitivity calculations. We evaluated the influence of the local finite element size on the maximum stress at its location for both analyzed variants. The discretization error was considered acceptable when the change in maximum equivalent stress was less than 5% upon halving the element size, confirming adequate resolution for the key output variables. The final mesh was therefore selected as an optimal compromise between computational accuracy and efficiency, taking into account the available hardware resources and acceptable computation time [15]. Computations and meshing were performed on a workstation equipped with an AMD Ryzen processor (3.8 GHz, 24 cores), 256 GB DDR4 RAM, NVIDIA T1000 GPU, 4 TB SSD storage, and running Windows Server operating system.

### Material models

All materials were modeled as linearly elastic, homogeneous, and isotropic, based on their Young’s modulus and Poisson’s ratio. Material properties were carefully selected from relevant prior studies (Table 1). Based on prior research [23], the allograft was considered to be as mechanically competent as healthy cortical bone tissue.

**Table 1:** The material properties for each part of the assembly

Part	Material	E (MPa)	References
Fixation plate, screws	Stainless steel (316L)	195 000	[24]
Femur, allograft	Cortical bone	17 600	[25, 26]
Femur	Cancellous bone	350	[26]
Tibia	Cortical bone	17 000	[27]
Tibia	Cancellous bone	500	[27]
Bone cement (PMMA)	PMAA (Palacos®)	2 891	[28, 29]
Polyethylene components	UHMWPE	670	[30]
Cartilages	Cartilage	12	[25]
Menisci	Menisci	59	[31]

### Loads and boundary conditions

The load model represents the static equilibrium of a person standing upright on both legs. The body weight is assumed to be 80 kg, corresponding to an applied force of 400 N acting on the surface of the femoral head along the mechanical axis of the lower limb. A fixed boundary condition, restricting all displacements, is applied at the distal end of the tibia. Because the model does not include the pelvis and surrounding musculature, an additional boundary constraint is introduced at a single node on the femoral head to prevent motion in the anterior–posterior direction. Figure 2 illustrates the applied boundary conditions. Ligamentous structures were included only in the OCH model because this reconstruction relies on soft-tissue restoration to achieve knee stability. In standard osteoarticular allograft reconstruction, stability may be restored using several techniques, including reattachment of host soft tissues to donor allograft soft tissues or to their anatomical insertion sites, and fixation with transosseous tunnels, anchors, or synthetic reconstruction material such as a Trevira tube. Accordingly, the ACL, MCL, and LCL were represented by tension-only spring elements in the OCH model. In contrast, the APC model incorporated a revision rotating-hinge knee prosthesis, in which primary coronal and sagittal plane stability is provided by the intrinsic mechanical constraint of the hinge mechanism. Additional ligamentous spring elements were therefore not included in the APC model, as this would not represent the clinical construct and could artificially increase the overall constraint of the reconstruction. It was implemented in ANSYS using three spring elements positioned at their corresponding anatomical attachment sites. The springs were defined to act in tension only, simulated in ANSYS using the LINK180 element type set to “tension-only” behavior. Each ligament was modelled by assigning a specific stiffness k [N/mm]: ACL—83.15 N/mm, MCL—91.25 N/mm, and LCL—72.22 N/mm [32].

All interfaces between the screws, cortical bone, and cancellous bone were modelled as frictional contacts. The screw thread geometry model was used, accurately reflecting the real shape. Frictional contact conditions were also defined between all bone tissues and the PMMA material, as well as at the interface between the original bone and the allograft. In the knee joint, frictional contact was applied between the articular cartilages and the menisci. The corresponding friction coefficients are listed in Table 2.

**Table 2:** The applied friction coefficients to the surface pairs

Contact surfaces	Friction coefficient [–]	References
316L–cortical bone	0.37	[33]
316L–cancellous bone	0.25	[34]
Cortical bone–cortical bone (allograft)	0.58	[35]
Cortical bone (allograft)–cancellous bone	0.58	[35]
Cortical bone–PMMA	0.3	[36]
Cancellous bone–PMMA	0.60	[37]
Cartilage–cartilage	0.02	[38]
Menisci-cartilage	0.02	[38]

## Results

### Equivalent elastic strain

**Fig. 3:**
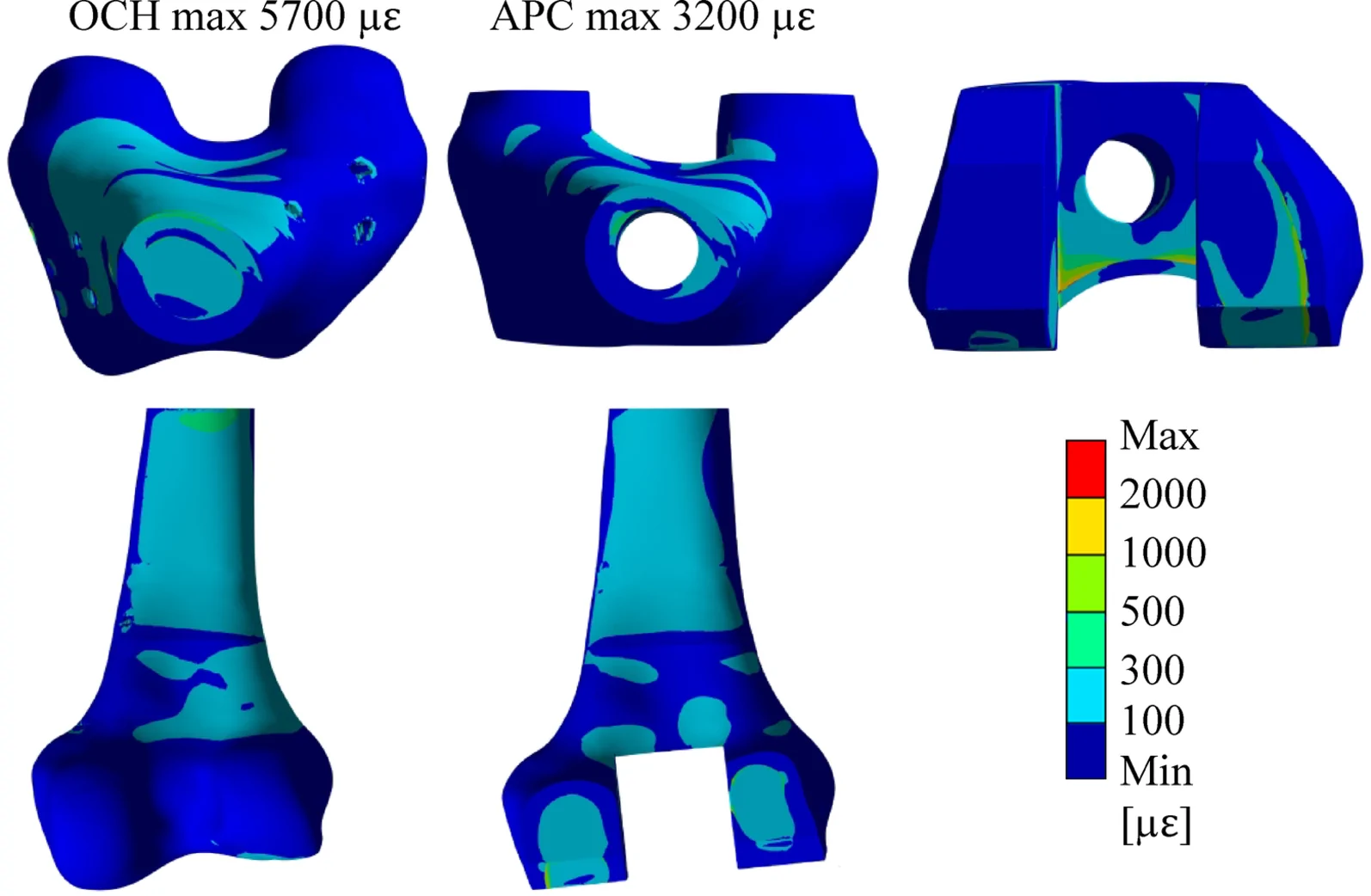
Equivalent von Mises strain distribution in the allograft for both APC and OCH models in transversal and posterior view

Figure 3 shows the equivalent von Mises microstrain distribution in different views. Microstrain (µε) quantifies relative deformation, where a value of 1000 µε represents a 0.1% change in length. Microstrain values were categorized according to the mechanostat concept [39, 40].

In the OCH model, peak strain values are concentrated in the immediate vicinity of the thread–bone interface, where they locally reach up to 5700 µε. Elevated strains are also observed along the interface with the host bone; however, these values remain below 800 µε. In the APC model, the maximum recorded strain is 3200 µε, localized at the intercondylar notch near the cancellous–cortical bone interface model.

**Fig. 4:**
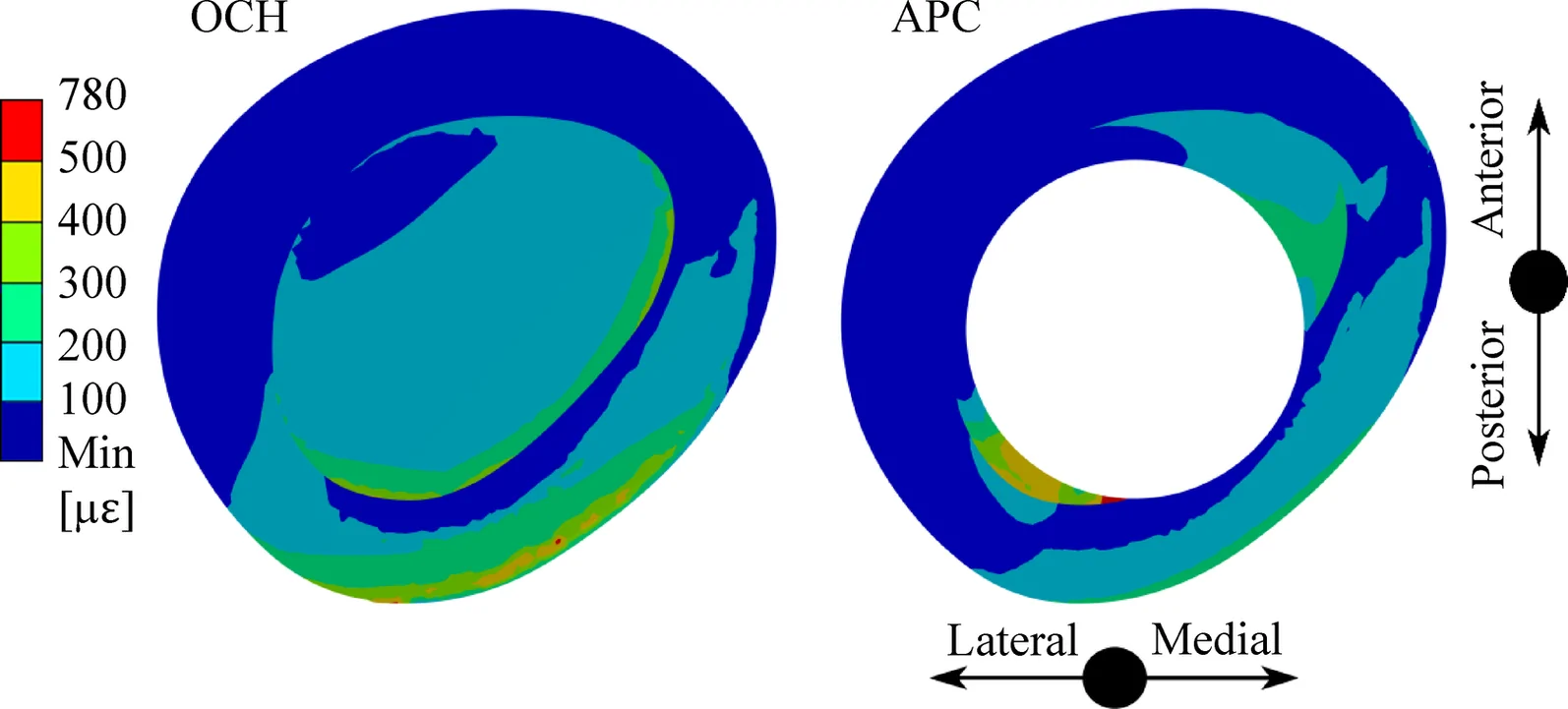
Equivalent von Mises strain distribution in the host–graft bone interface for both APC and OCH models

Figure 4 illustrates the host–graft bone interface in detail, with equivalent strain mapped on the cortical and cancellous bone models. In both models, the equivalent strain does not exceed 780 µε.

### Volumetric values of equivalent elastic strain

**Fig. 5:**
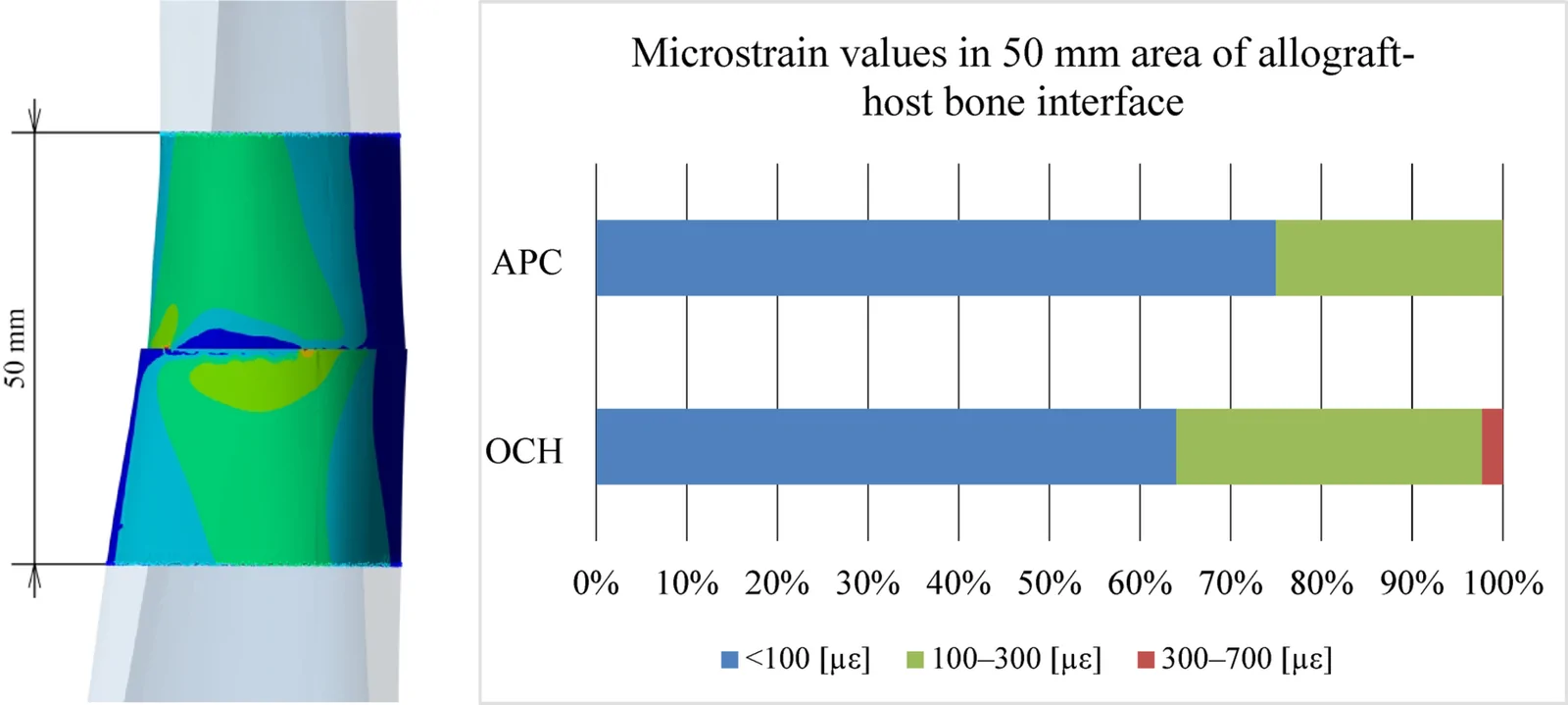
Equivalent von Mises strain distribution in the host–graft bone interface for both APC and OCH models

Figure 5 presents the microstrain values for each model as a representative contour plot and the corresponding volume profile. Strains were evaluated within a volume of interest in the cortical bone model, defined as a region extending 25 mm on either side of the host–graft bone interface, measured along the longitudinal axis. In the OCH model, strains below 100 µε account for 64.0% of the evaluated volume, compared with 75.0% in the APC model. Conversely, the remaining 36.0% of the OCH volume exhibits strains above 100 µε, reaching up to 700 µε. In the APC model, the remaining 25.0% of the volume shows strains above 100 µε, with less than 0.1% of the volume exceeding 300 µε.

### Interfragmentary motion and sliding distance of the allograft

To analyze interfragmentary motion (IFM) between the host bone and allograft, we adopted the comprehensive approach by Sun et al. [14] for similar orthopedic reconstructions. The Python 3.10.11-based IFM-Cal program was used to quantify IFM and sliding distances, following data preparation in the Ansys environment. This method provides accurate, reliable results for our biomechanical evaluation. The IFM distance quantifies axial and tangential motion of the analyzed components relative to a local coordinate system centered at the midpoint of the host bone surface (per IFM-Cal program guidelines). The sliding distance captures only the tangential motion component.

**Fig. 6:**
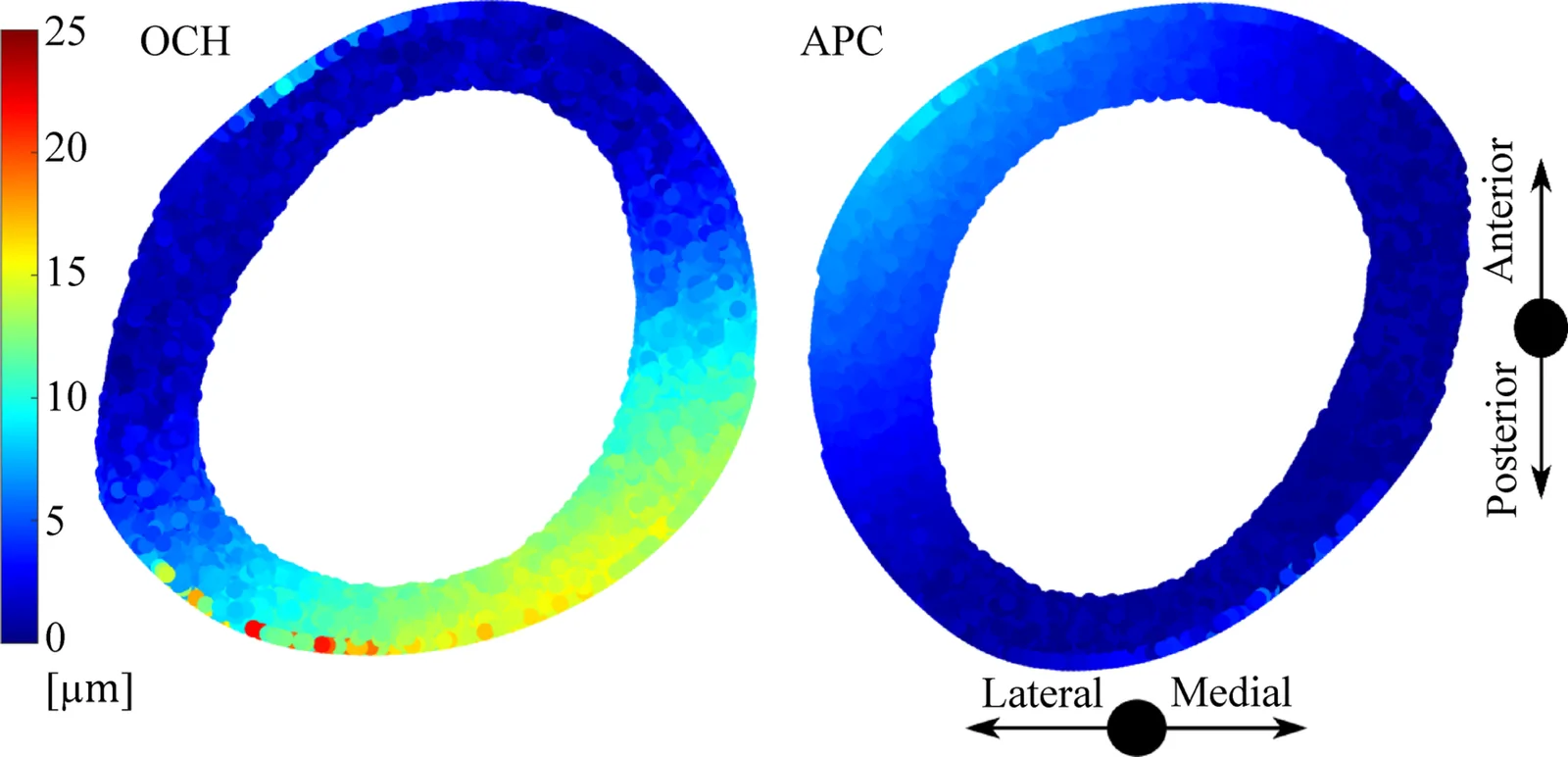
IFM distribution for both analyzed variants

Figure 6 illustrates the IFM values along the host–graft bone interface. The OCH model demonstrates the greatest IFM distance of 25 µm, whereas the APC model reaches only 10 µm, with the maxima occurring at different regions of the interface. Overall, the median IFM distance for the OCH model is 6.5 ± 5.8 µm, while for the APC model it is 2.5 ± 2.4 µm.

**Fig. 7:**
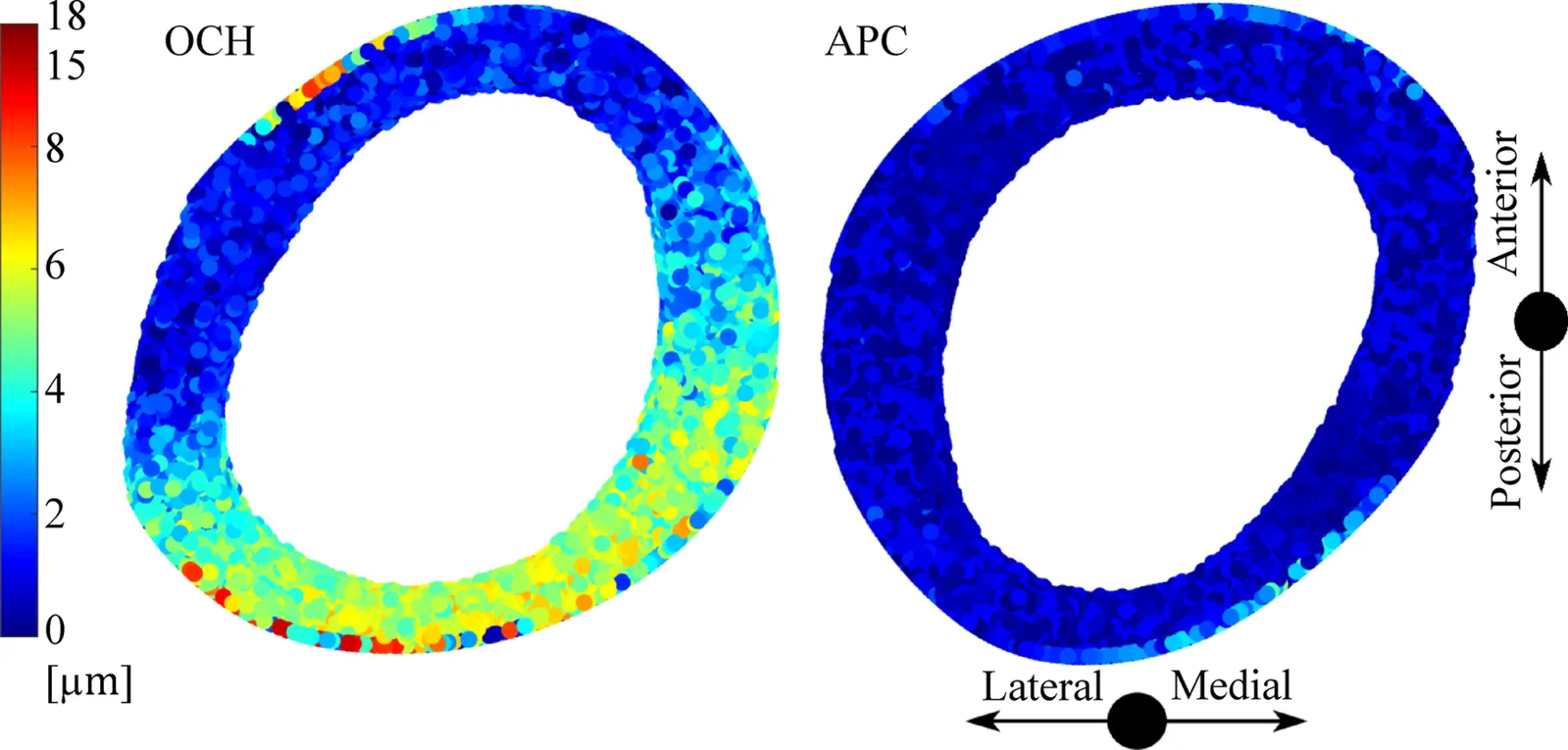
Sliding distance distribution for both analyzed variants

Figure 7 shows the SD values along the host–graft bone interface. The OCH model reaches a maximum SD of 18 µm, while the APC model reaches only 6 µm, and these peaks occur at different locations. The median SD distance is 3.5 ± 3.0 µm for the OCH model and 0.6 ± 0.9 µm for the APC model.

### Equivalent elastic stress

**Fig. 8:**
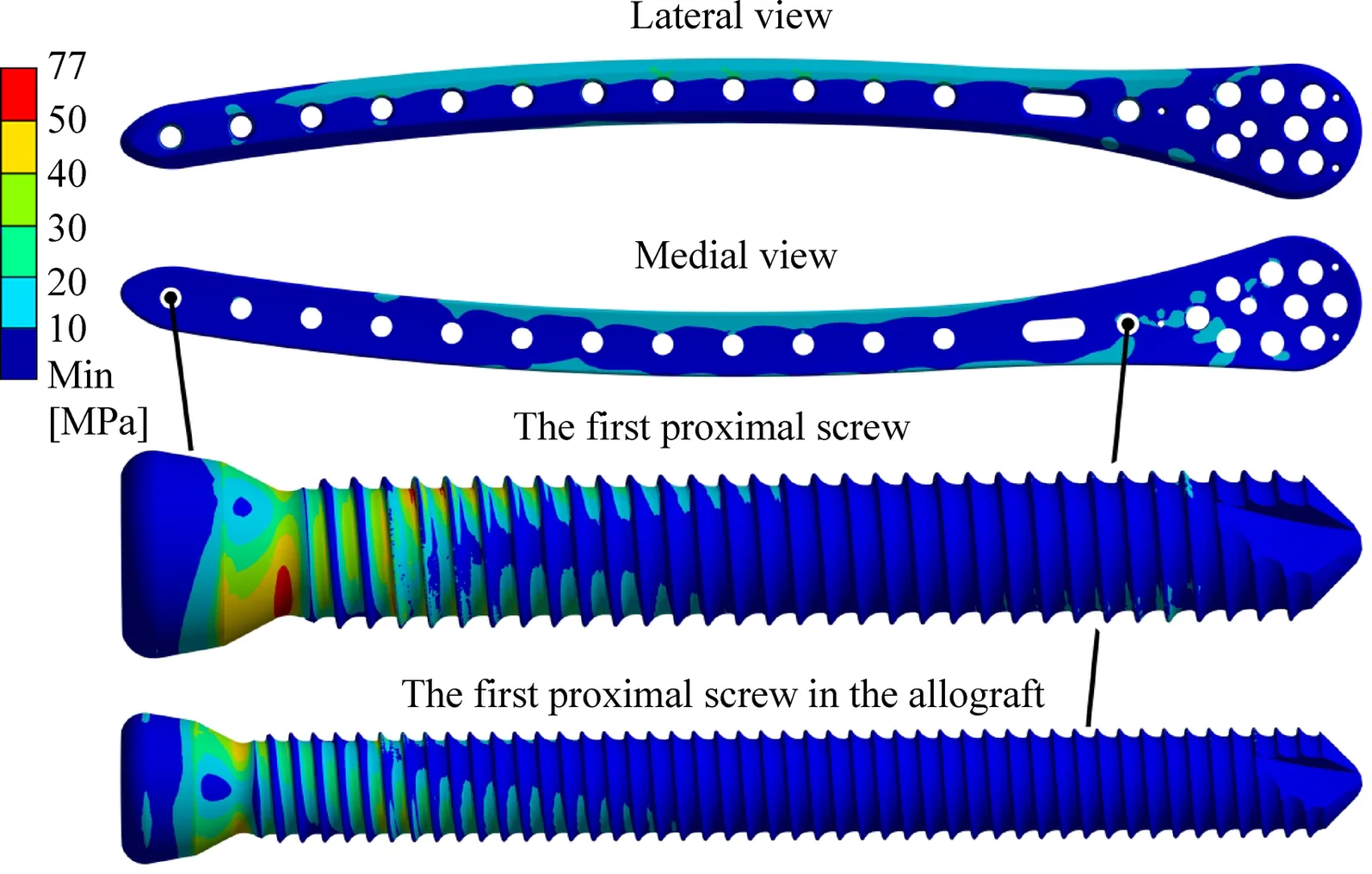
Equivalent von Mises stress distribution in the distal femoral plate and two most loaded screws

Figure 8 presents the equivalent (von Mises) stress in the DFP plate and in the two most highly loaded screws, located at the most proximal positions in the host bone and in the allograft. The DFP plate reaches a maximum stress of 43 MPa at the internal conical surface around the first proximal screw head, while stresses along most of the plate length remain within 20–30 MPa. The screw exhibits peak stress of 77 MPa at the neck region and approximately 70 MPa in the first few threads. The second most loaded screw is the most proximal screw within the allograft, with a maximum neck stress of 60 MPa.

**Fig. 9:**
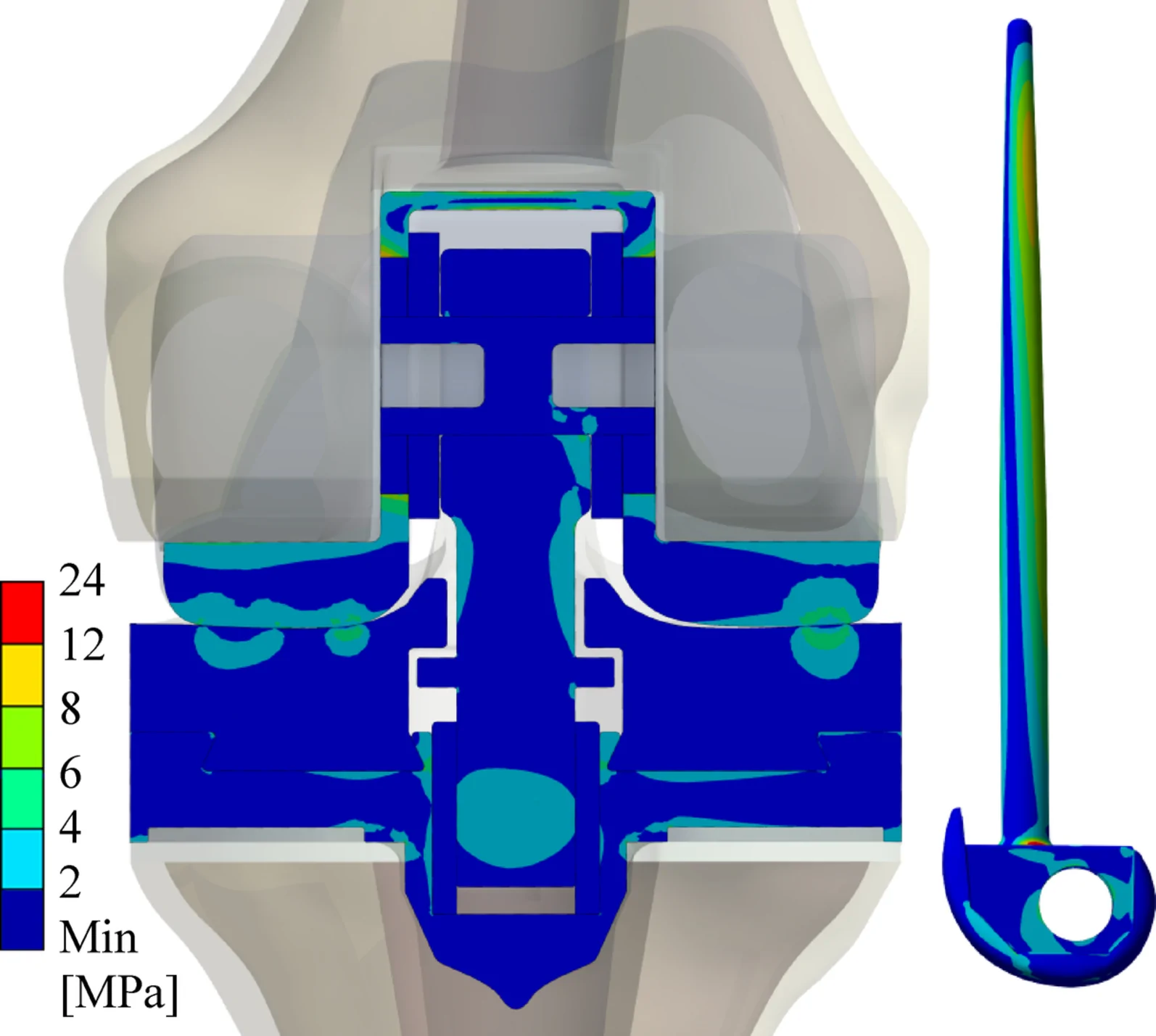
Equivalent von Mises stress distribution in the cut of revision rotating-hinge knee prosthesis assembly, and detail for femoral part

Figure 9 presents the equivalent (von Mises) stress distribution in the revision rotating-hinge knee prosthesis assembly. The highest recorded stress is 24 MPa, located in the root radius of the femoral stem, and similar higher values are located under the intercondylar notch. The highest stress in the polyethylene component was 8 MPa in contact with femoral component and tibial insert.

### Total deformation

**Fig. 10:**
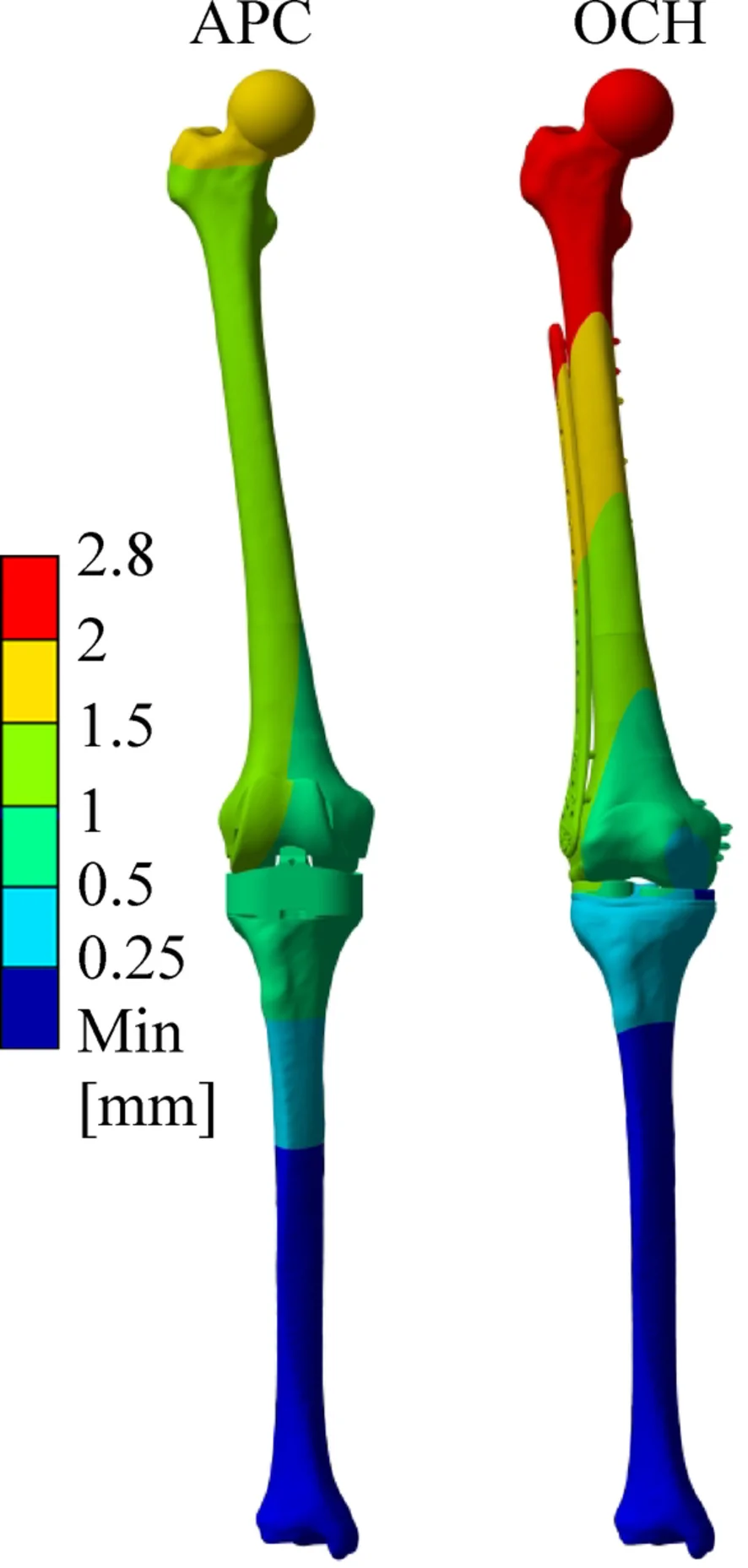
Total deformation contour plot for APC and OCH variants

To express the rigidity of both reconstructions, we analyzed the total deformation contour plots shown in Fig. 10. The APC reconstruction exhibits a maximum deformation of 1.8 mm, whereas the OCH reconstruction reaches 2.8 mm, indicating a less rigid construct.

## Discussion

The present finite element analysis provides a mechanistic explanation for the well-recognized clinical failure modes of both OCH and APC following distal femoral tumor resection. Although both reconstruction techniques restore bone stock, their mechanical environments at the host–graft junction differ substantially. These differences help clarify the origin of hardware breakage, malunion, and allograft resorption [22]. These findings offer a unifying biomechanical explanation for the persistent junctional complications observed in both techniques. They demonstrate that failure is not solely a biological phenomenon but is strongly governed by construct-specific load transfer characteristics. To our knowledge, this is the first finite element study directly comparing osteoarticular allograft and APC reconstructions under identical loading conditions, resection length, and allograft length. We also controlled the same position and magnitude of the acting force on the femoral head and fixed displacements at the distal end of the tibia. We quantitatively characterized interfacial strain, micromotion, and stress distribution as determinants of mechanical failure. Importantly, the rationale of the present study was not to determine the overall superiority of one reconstruction technique over the other. Both OCH and APC are established limb-salvage strategies with distinct clinical indications, advantages, and limitations [7, 9]. Rather, our objective was to isolate and compare the mechanical environments generated by each construct under identical loading conditions, resection length, and allograft length in order to better understand their characteristic failure mechanisms.

The equivalent elastic strain analysis highlights fundamentally different mechanical environments within the structural allograft in OCH and APC reconstructions, offering a direct explanation for their distinct failure patterns. In the OCH model, strain was more widely distributed. A greater proportion of the graft was exposed to values within the osteogenic window, indicating effective load sharing but also increased mechanical demand on the cortical structure. Structural allograft fracture should also be considered in the context of progressive fatigue damage. Massive allografts have limited remodeling capacity and may remain biologically compromised for a prolonged period. In APC reconstruction, load is partly transferred through the prosthetic stem, whereas in OCH reconstruction, the graft is exposed to more physiological but repetitive load sharing. Therefore, both constructs may carry a risk of fatigue-related graft failure through different mechanisms. Because the present model used a static early postoperative load, it cannot directly predict fatigue fracture over time; however, the observed construct-specific strain and stress patterns may help explain different fatigue-risk environments. Such elevated strain regions may act as precursors to fatigue damage accumulation and ultimately allograft fracture under increased loading [41, 42]. Conversely, the APC configuration demonstrated a clear shift toward predominantly low strain levels (< 100 µε) at the host–graft junction, reflecting load transfer through the rigid stem and cement mantle rather than through the graft itself. While this strain reduction may decrease the risk of structural fracture, it simultaneously suggests mechanical under-stimulation consistent with stress shielding, which may impair biological incorporation and predispose to delayed union or progressive graft resorption [43, 44].

The equivalent elastic stress results align with the clinically observed pattern that hardware failure occurs more frequently after OCH reconstruction than after APC [45]. In our models, the OCH reconstruction demonstrated higher stress concentrations within the fixation hardware (peak equivalent stress ≈  77 MPa, with extensive regions exceeding 30 MPa), suggesting an increased mechanical demand on the stabilizing construct even under the intentionally low loading conditions simulating the early postoperative phase. Considering the yield strength of 316L stainless steel (690 MPa), these stress magnitudes remain well below the material’s plastic limit [46]. The analysis assumes linear elastic behavior of material models, and there are no significant changes in contact states during the load process. These stresses would be expected to increase proportionally with higher functional loads (up to the elastic limit of the materials). This would further elevate fatigue risk and thereby provide a plausible mechanical explanation for the higher incidence of hardware breakage reported clinically in the OCH series. In contrast, the APC configuration exhibited a peak equivalent hardware stress of 24 MPa under the same loading conditions, indicating a distinct load-transfer pattern through the rigid stem–cement–prosthesis system. These results illustrate distinct mechanical behaviors rather than the superiority of one construct.

Prior experimental and computational work has shown that micromotion exceeding the low hundreds of micrometers, particularly when dominated by shear, may impair direct bone healing and favor fibrous tissue formation [47, 48]. IFM and sliding distance at the host–graft junction provide a mechanical framework for understanding the reported incidence of malunion and delayed union in distal femoral reconstructions. In the present analysis, the OCH model exhibited higher IFM values (peak 25 µm; median 6.5 ± 5.8 µm) and greater sliding distances at the junction (peak 18 µm; median 3.5 ± 3.0 µm) than the APC model (peak IFM 10 µm; median 2.5 ± 2.4 µm; sliding distance peak 6 µm; median 0.6 ± 0.9 µm). Accordingly, the APC configuration showed approximately 60% lower median IFM and about 80% lower median sliding distance compared with the OCH reconstruction. The resulting micromotion metrics are in good agreement with previous computational results from a comparable study, supporting the present model [15]. Although all values remained within a range compatible with early postoperative protection under the simulated 400 N load, the relative increase in micromotion and tangential displacement in OCH suggests a mechanically less constrained interface. Under higher physiological loading, this could contribute to asymmetric healing or progressive malalignment. Conversely, APC exhibited lower overall micromotion, indicating greater immediate stability. However, excessive rigidity may also reduce beneficial axial stimulation for callus formation. Therefore, while our results do not demonstrate a definitive mechanical cause of malunion, they indicate relative differences in IFM magnitude and sliding distance between constructs, which may contribute to distinct junctional mechanical environments and should be interpreted cautiously in relation to clinical healing patterns.

Several limitations of this study should be acknowledged. First, the applied loading condition represents a simplified early postoperative scenario corresponding to the recommended protected weight-bearing regime and was restricted to purely axial loading. Second, construct alignment, cement mantle, and host–graft contact were idealized, assuming perfect apposition without interfacial gaps, whereas in clinical reality, small mismatches or incomplete contact at the junction may alter local stress or strain distributions and higher IFM values. Third, although patient-specific geometries were used, the analysis is based on representative datasets and therefore may not capture the full variability in anatomy, bone quality, or surgical execution encountered in clinical practice. Fourth, the findings are specific to the particular fixation plate system and revision rotating-hinge knee prosthesis modeled in this study, and different implant designs or material properties could influence the mechanical response. Finally, the model was not directly experimentally validated; however, the predicted sliding distances are in good agreement with previous computational results from a similar study, supporting the present model [15]. Despite these limitations, the controlled numerical framework allows isolation of construct-specific mechanical behavior under identical allograft size and load conditions, providing mechanistic insight into complication patterns following distal femoral reconstruction.

## Conclusions

Under identical geometry and early postoperative loading conditions, OCH and APC reconstructions demonstrated distinct load-transfer patterns. OCH was characterized by greater graft strain, whereas APC demonstrated reduced graft strain. Hardware stress in the APC appeared consistent with the redistribution of stress through the stem. OCH also exhibited higher interfragmentary motion and sliding distance, while APC demonstrated greater immediate stability with lower micromotion at the host–graft interface. Rather than indicating superiority, the results highlight that each reconstruction is governed by a characteristic mechanical environment that may influence its failure pattern.

## Data Availability

The datasets used and/or analysed during the current study are available from the corresponding author on reasonable request.

## Abbreviations

OCH: Osteoarticular allograft
APC: Allograft–prosthesis composite
IFM: Interfragmentary motion
CT: Computed tomography
STL: Standard Tessellation Language
DFP: Distal femoral plate
PMMA: Polymethylmethacrylate
UHMWPE: Ultra-high-molecular-weight polyethylene
ACL: Anterior cruciate ligament
MCL: Medial collateral ligament
LCL: Lateral collateral ligament
AO: Arbeitsgemeinschaft für Osteosynthesefragen
